# Experimentally induced subclinical mastitis: are lipopolysaccharide and lipoteichoic acid eliciting similar pain responses?

**DOI:** 10.1186/s13028-017-0306-z

**Published:** 2017-06-14

**Authors:** Annalisa Elena Jolanda Giovannini, Bart Henricus Philippus van den Borne, Samantha Kay Wall, Olga Wellnitz, Rupert Max Bruckmaier, Claudia Spadavecchia

**Affiliations:** 1 Department of Clinical Veterinary Medicine, Anesthesiology and Pain Therapy Section, Vetsuisse Faculty, University of Berne, Laenggassstrasse 124, 3012 Berne, Switzerland; 2 Veterinary Public Health Institute, Vetsuisse Faculty, University of Berne, Schwarzenburgstrasse 155, 3097 Liebefeld, Switzerland; 3 Veterinary Physiology, Vetsuisse Faculty, University of Berne, Bremgartenstrasse 109a, 3012 Berne, Switzerland

**Keywords:** Mastitis, Lipoteichoic acid, Lipopolysaccharide, Dairy cow, Pain

## Abstract

**Background:**

Pain accompanying mastitis has gained attention recently as a relevant welfare compromising aspect of disease. Adequate pain recognition and therapy are necessary in the context of a modern and ethically acceptable dairy care. For research purposes mastitis is often induced by intramammary infusion of immunogenic bacterial cell wall components. Lipopolysaccharide (LPS) from *Escherichia coli* and lipoteichoic acid (LTA) from *Staphylococcus aureus* are commonly administered to this end. While the immune response to specific immunogenic components has been well characterized, not much is known about their role on the expression of pain indicators. The aim of this study was to trial the effects of an intramammary challenge of LTA or LPS on the degree of pain and discomfort as indicated by both physiological and behavioral variables in cows. The hypothesis was that a similar degree of pain can be identified in LTA as well as in LPS induced mastitis.

**Results:**

On the challenge day, compared to pre-challenge, total pain index increased for all treatment groups (LPS; LTA and control), the LPS group having significantly higher values than the control group (P = 0.01). Similarly, pain visual analogue scale (VAS) increased significantly in all cows following treatment on the challenge day. Furthermore, compared to baseline, higher VAS were found 3, 4 and 5 h after the challenge in cows of the LPS group (P_3h, 4h_ < 0.001 and P_5h_ = 0.001) and 7 h after the challenge in cows of the LTA group (P_7h_ = 0.002). In the control group, VAS was higher 5 h after the challenge (P_5h_ = 0.001). On the challenge day, udder edema was higher in the LPS than in the control group (P = 0.007). Furthermore, 4 h after the challenge, milk cortisol was significantly higher than at baseline in the LPS group (P < 0.001).

**Conclusions:**

When administered at equipotent doses targeting a standard somatic cell count increase, intramammary LPS seems to be accompanied by a higher degree of pain and discomfort than LTA, as suggested by the modifications of the outcome variables total pain index, VAS, udder edema and milk cortisol.

**Electronic supplementary material:**

The online version of this article (doi:10.1186/s13028-017-0306-z) contains supplementary material, which is available to authorized users.

## Background

Bovine bacterial mastitis has been increasingly recognized as a detrimental disease in dairy herds, leading to major economic losses and premature culling [1]. Mastitis-related pain and discomfort can severely compromise animal welfare and deserve to be specifically addressed [2]. In the last decade several indicators have been proposed to recognize and quantify pain in bovine mastitis. Physiological parameters such as heart rate, respiratory rate and temperature [2], modifications of postural and ingestive behaviors [3, 4], expression of active behavior like stepping, kicking and limb lifting [5, 6] and alteration in nociceptive thresholds [7, 8] have been all applied to this end.

Most of the recent research aiming at characterizing the physiological, immunological and behavioral response to pathogenic invasion of the mammary gland has been conducted on experimentally induced mastitis [6, 7, 9, 10]. Two major immunogenic cell wall components, lipoteichoic acid (LTA) and lipopolysaccharide (LPS), deriving from *Staphylococcus aureus* and *Escherichia coli* respectively, are commonly infused in the bovine mammary gland to elicit experimental disease [10–13]. In spontaneous mastitis, *S. aureus* tend to induce a rather chronic subclinical disease, while *E. coli* is typically isolated in acute clinical cases. Whereas a pathogen-specific immune response of the mammary gland in LPS and LTA-induced mastitis has been confirmed by several studies [10, 13, 14], not much is known about the role of these specific immunogenic components on the expression of clinical pain indicators. Indeed, pain-related physiological and behavioral alterations have been widely investigated in LPS [3, 4, 7] but not in LTA induced mastitis. As *S. aureus* mammary infections lead to breast pain in humans [15], we hypothesized that a similar degree of pain can be identified in LTA as well as in LPS induced mastitis in lactating dairy cows.

The main aim of this study was to trial the effects of an intramammary challenge of LTA or LPS on the degree of pain and discomfort as indicated by both physiological and behavioral variables in cows.

## Methods

The study was approved by the Cantonal Committee for Animal Experimentation, Fribourg, Switzerland (FR16/13) and all experimental procedures followed the Swiss law of animal protection.

### Animals

Sixteen lactating dairy cows (12 Holstein and 4 Swiss Fleckvieh) in mid lactation (mean DIM = 202 ± 88), involved in a larger immunological study [13], were enrolled in this experiment. Parities of experimental cows ranged from 1 to 4 (average parity = 2.6) and cows were producing >15 l of milk/day (mean milk yield = 18.4 ± 3.9 l/day). They were housed in a stanchion barn and were kept in single rowed tie-stalls (width 250 cm, length 200 cm) on rubber mats bedded with wood shavings and straw. Hay was available ad libitum, as well as water (through individual water bowls); concentrate was fed according to individual production levels. Cows were machine milked at 0530 and 1600 h. Before the trial, animals were allowed to acclimatize to the new environment for at least 1 week.

Inclusion criteria for the experiment were: body condition score (BCS) of 2.5–3, healthy based on clinical examination, negative glutaraldehyde test, normal hematology and blood chemistry, milk somatic cell count (SCC) of each quarter <150 × 10^3^ cells/ml and negative milk bacteriology.

### Experimental design

The study was designed as a prospective, blinded, controlled experimental trial. Cows were randomly allocated to one of 3 treatment groups: LPS group (n = 6), LTA group (n = 6) and control group (C group, n = 4). For each animal, the trial started 24 h before the intramammary challenge and ended 26 h later. A maximum of two cows were studied at once. On the day preceding the challenge (control day), baseline outcome parameters were measured over 8 h and intramammary infusion, milk and blood sampling were simulated at the same time points as during the challenge day. Once terminated the 8 h data collection, a catheter (length 105 mm −Ø 1.9 × 2.4 mm −13 G, Vygon, Ecouten, France) was introduced in the jugular vein and a liver biopsy was performed. On the challenge day, immediately after morning milking and following aseptic preparation of the teat, animals received an intramammary infusion containing the assigned treatment through a sterile teat cannula (length 100 mm, −Ø 22 mm, Delvo, Switzerland; time 0). The treatments consisted of 0.2 µg LPS (from *E. coli* serotype O26:B6, Sigma-Aldrich, St. Louis, MO, USA) diluted in 10 ml of 0.9% sterile saline for group LPS, 20 µg LTA (from *S. aureus*, Sigma-Aldrich, St. Louis, MO, USA) diluted in 10 ml of 0.9% sterile saline for group LTA, and 10 ml of sterile saline 0.9% alone for group C. Dosages of LPS and LTA were chosen to induce a similar SCC increase. For each animal 2 randomly allocated quarters (one front and one hind) were infused according to the treatment, while the other 2 received an equivalent volume of sterile 0.9% saline. On the challenge day, outcome parameters were collected over 8 h starting from time 0. Once terminated the data collection, a liver and two udder biopsies on the hind quarters were performed. All biopsies, performed for the purpose of the immunological study [13] in all groups, were taken in unsedated but physically restrained cows after skin desensitization with 10 ml of lidocaine (Lidocaine 2%, Streuli Pharma AG, Switzerland). A last clinical examination and recording of outcome parameters was performed 26 h after the challenge. At this time point, rescue analgesia (3 mg/kg ketoprofen IV; Rifen 10%, Streuli Pharma AG, Switzerland) was provided if pain visual analogue scale (VAS) score >3 (for VAS description see below). The investigator performing clinical pain assessment (AEJG) was blinded to the treatment.

### Outcome parameters

#### Physiological parameters

The following physiological parameters were recorded at hourly intervals: heart rate, respiratory rate and rectal temperature. Heart rate was continuously monitored using a portable heart rate monitoring system (Polar RS800CX, Polar Electro Europe BV, Switzerland) inserted in a girth positioned around the cow’s chest before the trial started. Respiratory rate was evaluated through visual detection of costoabdominal distension, while rectal temperature was measured with a digital thermometer.

#### Ingestive and postural behavior

Time spent eating, ruminating, and lying were automatically recorded for 8 h during both the control and the challenge day using a recently validated monitoring system (Rumiwatch, Itin+Hoch GmbH, Switzerland) [16]. On cows standing with parallel hind limbs, hock-to-hock distance was measured every hour with a centimeter-scaled rolling tape as the distance between the middle points of each calcaneal tuberosity [17].

#### Pain scoring

For pain assessment, a mastitis-specific multidimensional pain scoring system was designed. The scoring system was organized in two main categories of symptoms, general and local, which were further divided in a total of eight sub-categories. Each sub-category was scored using simple numerical rating scales except for the sub-category postural behavior, for which a score of 1 was assigned to each of the observed manifestations. At each specific time point, assigned scores were summed to obtain the total pain index, with a maximum possible value of 42 (Table 1). Furthermore, pain severity was scored using a dynamic-interactive VAS on a 100 mm line (0 meaning no pain, 100 mm meaning the worst possible pain) [18].

**Table 1:** Multidimensional pain scoring system, including general and local items, divided in 8 sub-categories

Category	Sub-category	Manifestation	Assigned value
General	General subjective assessment	No signs of pain	0
			1
			2
			3
		Signs of severe pain	4
	Postural behavior	Low, asymmetric ears	1
		Corrugated upper eyelids	1
		Open nostrils	1
		Restless	1
		Apathy	1
		Wide hind limbs	1
		Other (specify)	1
	Interactive behavior	Interest	0
			1
		No interest	2
	Response to food	Appetite	0
			1
		No appetite at all	2
	Sacrum position	Normal	0
		Downward with arched back	1
	Reaction to back palpation	No reaction	0
			1
		Strong reaction	2
Local	Udder edema	FL	0-1-2-3-4
		FR	0-1-2-3-4
		HL	0-1-2-3-4
		HR	0-1-2-3-4
		0 = no swelling, 4 = very severe swelling	
	Udder palpation	FL	0-1-2
		FR	0-1-2
		HL	0-1-2
		HR	0-1-2
		0 = no reaction, 1 = mild reaction (tail flicking, limb lifting), 2 = strong reaction (kick, moving away)	
	Total pain index	Summation of scores	Max 42

Assigned scores are added to obtain the total pain index

*FL* front left quarter, *FR* front right quarter, *HL* hind left quarter, *HR* hind right quarter

#### Udder parameters

Udder edema and reaction to udder palpation were scored as part of the total pain index (local subcategory items, Table 1) but were also analyzed as separate variables.

Udder temperature was measured using an auto-calibrating thermic camera (InfraVet OptiRes D, VarioCAM, Infra Tech GmbH, Dresden, Germany). Two lateral views of the udder were recorded from a 50 cm distance and on animals left undisturbed for at least 10 min. Median udder surface temperature on predefined area was detected with a dedicated software (Exam Professional 5.8, InfraMedic GmbH, Germany), as previously described [19, 20].

A purpose-built digital hand-held pressure algometer with a contact surface of 0.5 cm^2^ was used to test the mechanical nociceptive thresholds (MNT) at the front quarters at 2 h intervals. Force was applied in caudal direction at a constant rate of 5 N/s perpendicularly to the udder surface, dorsally to the teat basis until an avoidance behavioral reaction was observed. Maximal peak force applicable was set at 24.6 N. Two measurements per quarter, taken at 60 s intervals, were averaged for analysis.

#### Laboratory analysis

During the challenge day, cortisol concentrations in milk and plasma aliquots, collected as described by Wall et al. [14] and stored at −20 °C, were measured at 2 h intervals with methods described elsewhere [21]. Somatic cell count (SCC) in fresh milk samples was measured hourly with a DeLaval cell counter (DCC, DeLaval International AB, Tumba, Sweden) according to the manufacturer’s protocol.

### Statistical analysis

Statistical analysis of outcome parameters (see Additional file 1) was performed using hierarchical regression models built with PROC GLIMMIX within SAS 9.4 (SAS Institute, Inc., Cary, NC). Cows’ activities eating, ruminating and laying were expressed as a percentage over the total recording time and aggregated at day level. The quarter outcome variable “reaction to udder palpation” was also aggregated at day level and represented whether any of the measurements on that day had a value ≥1. Presence of udder edema was classified as yes or no applying a binomial distribution. The decimal logarithm of milk and plasma cortisol was taken for the data to follow a normal distribution. Cow level models included the covariates day, time, and treatment as fixed effects and all their 2- and 3-way interactions evaluated. The correlation structure with the best model fit was selected to correct for correlation among repeated measurements based on the Akaike Information Criterion. For quarter-level variables, the same modeling procedure was applied but “quarter” (infusion yes or no) was additionally evaluated as a fixed effect in the above-mentioned model. Moreover, a random intercept was added to correct for clustering of quarters within cows. After Bonferroni correction, significance was defined at P value <0.0045 for cow level outcome variables and at P < 0.01 for quarter level outcome variables to correct for multiple comparisons. Model fit of linear regression models was checked by evaluating normality and homoscedasticity of residuals. Reference categories of significant variables were changed to perform a post hoc analysis to determine significant differences in 2-way or 3-way interaction terms.

Descriptive analysis of cow demographics and spearman rank correlation coefficients between total pain index and VAS and between total pain index and SCC were calculated using SigmaPlot 12.0 (Systat Software GmbH, Germany).

## Results

**Fig. 1:**
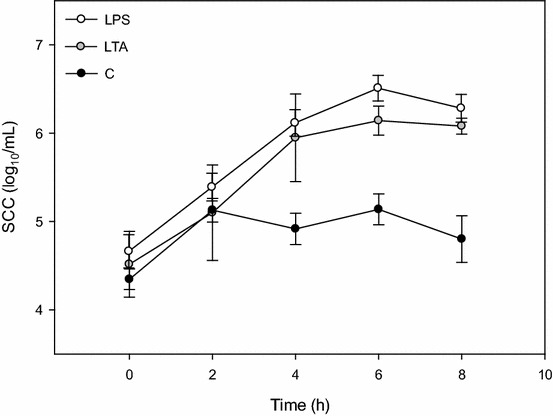
Somatic cell count of infused quarters. Changes in somatic cell count of infused quarters in the LPS (lipopolysaccharide) (*white circles*), LTA (lipoteichoic acid) (*grey circles*) and control groups (*black circles*) on the challenge day. Data are expressed as mean ± SEM

A total of 16 challenges were performed. One cow of the LTA group was excluded from statistical analysis because it had not responded to the challenge with the expected SCC increase. Thus, variables of 15 cases were evaluated. No significant differences were found among the groups concerning parity and DIM. Similar increases in SCC in infused quarters confirmed the equivalence of the LPS and LTA doses (Fig. 1).

All cows except one recovered well from the trial, as confirmed by the clinical examination 26 h after the challenge. A single cow in the LPS group received rescue analgesia at this time point.

### Physiological parameters

No statistical significant difference was detected among groups for the variables heart rate, respiratory rate and rectal temperature (Table 2).

**Table 2:** Heart rate (HR), respiratory rates (RR), rectal temperature (Rectal T) and hock-to-hock distance (H–H) measured in cows challenged with intramammary LPS (lipopolysaccharide), LTA (lipoteichoic acid) or saline (control group)

Parameter		Control day				Challenge day			
		2 h	4 h	6 h	8 h	2 h	4 h	6 h	8 h
HR (beats/min)									
LPS		76 (69–80)	71.5 (64–73)	71 (64–72)	74.5 (62–78)	69.5 (60–78)	71 (60–75)	71.5 (60–74)	70 (60–78)
LTA		69 (64–79)	71 (66–82)	71 (66–81)	71 (69–79)	67 (65–71)	67 (64–77)	76 (72–77)	70 (64–81)
Control		68 (62–73.5)	66.5 (59.5–72)	61.5 (57–71)	66 (61–70)	62.5 (57.5–67.5)	69 (62–70.5)	63.5 (58.5–66)	61.5 (57–67.5)
RR (breaths/min)									
LPS		30 (28–32)	32 (28–32)	32 (24–40)	32 (28–36)	28 (28–36)	32 (28–36)	30 (24–32)	30 (28–32)
LTA		32 (28–36)	32 (28–40)	36 (32–44)	32 (32–48)	32 (32–44)	32 (28–44)	44 (32–44)	36 (32–40)
Control		32 (28–34)	28 (22–38)	28 (26–42)	32 (30–36)	30 (24–36)	32 (28–38)	28 (24–40)	30 (28–40)
Rectal T (°C)									
LPS		38.1 (37.9–38.4)	38 (37.9–38.4)	38.2 (38.1–38.4)	38.1 (37.9–38.5)	38.2 (37.9–38.5)	38.3 (38.1–39.0)	38.4 (38.3–39.4)	38.8 (38.1–39.2)
LTA		38.3 (38.2–38.5)	38.2 (38–38.3)	38.3 (38.3–38.4)	38.5 (38–38.5)	38.3 (38.2–38.4)	38.4 (38.3–38.4)	38.5 (38.2–38.6)	38.7 (38.4–39.0)
Control		38.1 (37.9–38.3)	38.1 (37.9–38.3)	38.1 (37.9–38.3)	38.2 (38.1–38.3)	38.1 (38–38.1)	38.3 (38.1–38.3)	38.1 (38–38.2)	38.1 (38.1–38.3)
H–H (cm)									
LPS		22.5 (18–25)	21 (17–28)	20 (16–30)	26 (15–28)	22 (20–23)	23.5 (15–26)	20.5 (14–25)	23.5 (17–27)
LTA		23 (20–24)	22 (22–22)	22 (20–24)	22 (20–23)	18 (15–20)	20 (16–25)	23 (22–25)	23 (20–24)
Control		23 (17.5–24.5)	23.5 (17–26)	25 (19.5–28)	25 (20–27.5)	22.5 (17.5–25)	24 (23–26.5)	24.5 (24–27.5)	22 (19–26)

On the control day, the intramammary challenge, milk and blood sampling were simulated. Medians and interquartile ranges (IQR) at 2, 4, 6 and 8 h after the simulated challenge (control day)/challenge (challenge day) are reported

### Ingestive and postural behavior

Independently from the group, all cows spent significantly less time lying on the challenge day compared to the control day (P < 0.001). No significant difference among groups could be found for time spent eating and ruminating, although on the challenge day animals of the LPS group tended to eat less than those of the C group (P = 0.05) (Table 3). No differences in hock-to-hock distance were found among groups (Table 2).

**Table 3:** Total time spent lying, eating and ruminating by cows challenged with intramammary LPS (lipopolysaccharide), LTA (lipoteichoic acid) or saline (control group)

Parameter		Control day	Challenge day
Lying time (min)			
LPS		88.4 (48.8–118.5)	26.4 (18.2–31.6)
LTA		73.6 (51.4–92.9)	15.4 (0–41.7)
Control		110.6 (80.2–130.3)	39.1 (11.8–71.4)
Eating time (min)			
LPS		104.3 (82.2–110.7)	73.4 (58.9–89.9)
LTA		160 (103–183.3)	124 (68.3–178.9)
Control		146.2 (92.4–215)	167.9 (131.4–208)
Ruminating time (min)			
LPS		186.7 (83.5–201.1)	148.9 (12–146.9)
LTA		161.2 (140.5–221)	127 (85.1–146.9)
Control		142.1 (93.7–174.1)	109.1 (95.2–119.3)

On the control day, the intramammary challenge, milk and blood sampling were simulated. Medians and interquartile ranges are reported. Recordings were performed over 8 h on both the control and the challenge day

### Pain scoring

**Fig. 2:**
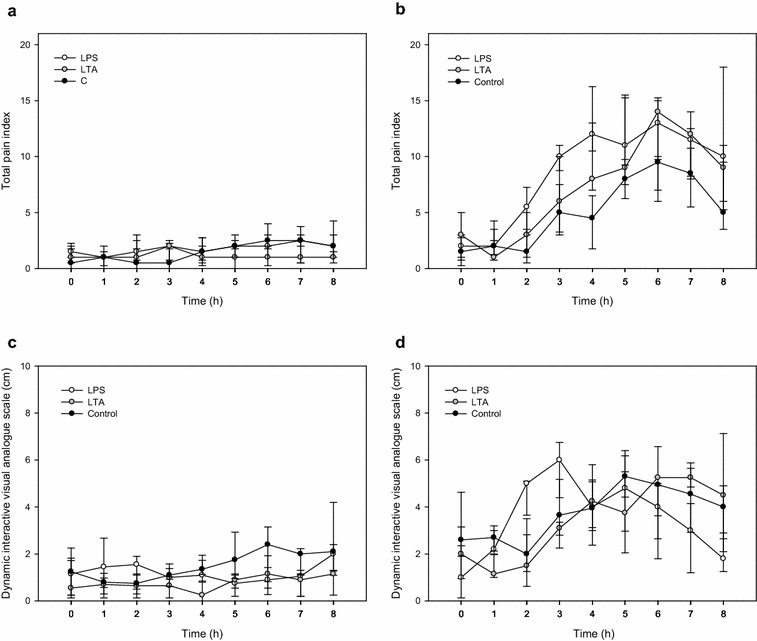
Pain scores on the control and challenge day. Total pain index (**a**, **b**) and dynamic interactive visual analogue scale (**c**, **d**) recorded in the LPS (lipopolysaccharide) (*white circles*), LTA (lipoteichoic acid) (*grey circles*) and control groups (*black circles*) during control day (**a**, **c**) and challenge day (**b**, **d**). Data are presented as medians and interquartile ranges

The variable total pain index (Fig. 2a, b) had a significant day × time interaction (P = 0.002). On the challenge day it increased for all 3 treatment groups, the LPS group having higher values than the control group (P = 0.01). Within the multidimensional pain scoring system, the mostly affected sub-categories were appetite, reaction to udder palpation and udder edema score. Results for udder variables are presented in the following paragraph.

**Fig. 3:**
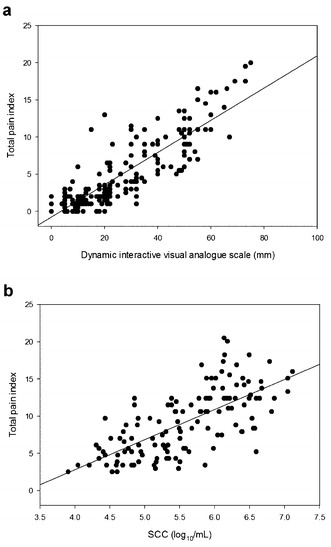
Correlation between 2 pain scoring methods, and between total pain index and somatic cell count. Spearman rank correlation between dynamic interactive visual analogue scale and total pain index (TPI) [**a**, ρ = 0.817 (*P* < 0.001)], and between somatic cell count (SCC) of infused quarters and TPI [**b**, ρ = 0.707 (*P* < 0.001)] in intramammary LPS (lipopolysaccharide), LTA (lipoteichoic acid) or saline challenged cows

Visual analogue scale data (Fig. 2c, d) showed a significant day × time × treatment interaction (P < 0.001). On the challenge day, higher VAS were found 3, 4 and 5 h after the challenge compared to t0 in cows of the LPS group (P_3h, 4h_ < 0.001 and P_5h_ = 0.001) and 7 h after the challenge in cows of the LTA group (P_7h_ = 0.002). In the control group, VAS was higher 5 h after the challenge (P_5h_ = 0.001). High correlation between total pain index and VAS (r = 0.817; P < 0.001), as well as between total pain index and SCC of infused quarters (r = 0.707; P < 0.001) were found (Fig. 3).

### Udder parameters

A treatment-independent significant day effect (P = 0.008) resulted for reaction to udder palpation, with higher presence of at least mild responses on the challenge day compared to the control day. Udder edema score had a significant day × time interaction (P < 0.001): it was increasingly detected over time during the challenge day whereas no such increase was observed on the control day. On the challenge day, udder edema was higher in the LPS than in the C group (P = 0.007) and tended to be higher in the LTA group (P = 0.016) than in the C group. Moreover, udder edema tended to be higher in infused than in control quarters (P = 0.013). For udder temperature, no day nor quarter effect was found but, compared to t0, significantly higher values were found 2 and 8 h after the challenge in the LPS group (P = 0.002 and P = 0.004, respectively), and 6 and 8 h after the challenge in the LTA group (P = 0.006 and P = 0.001, respectively). No changes over time were detected for the C group. No statistical significant difference was detected among groups for mechanical nociceptive thresholds (Table 4).

**Table 4:** Reaction score to udder palpation (Reaction score), udder edema score (Edema score), udder temperature (Udder T) and mechanical nociceptive thresholds (MNT) measured in infused quarters of cows challenged with intramammary LPS (lipopolysaccharide), LTA (lipoteichoic acid) or saline (control group)

Parameter		Control day				Challenge day			
		2 h	4 h	6 h	8 h	2 h	4 h	6 h	8 h
Reaction score									
LPS		0 (0–0)	0 (0–0)	0 (0–0)	0 (0–0)	0 (0–0)	0 (0–0)	0 (0–0)	0 (0–0)
LTA		0 (0–0)	0 (0–0)	0 (0–0)	0 (0–0)	0 (0–0)	0 (0–0)	0 (0–0)	0 (0–0)
Control		0 (0–0)	0 (0–0)	0 (0–0)	0 (0–0)	0 (0–0)	0 (0–0)	0 (0–0)	0 (0–0)
Edema score									
LPS		0 (0–1)	0 (0–0)	0 (0–0)	0 (0–0)	1 (1–1)	2.5 (2–3)	2 (1–3)	1.5 (1–2)
LTA		0 (0–1)	0 (0–1)	0 (0–1)	0 (0–1)	0 (0–0.2)	1 (0.75–2)	1 (0.75–2.25)	1 (0.75–1.5)
Control		0 (0–0)	0 (0–0.5)	0 (0–0)	0 (0–0)	0 (0–0)	0 (0–0)	0 (0–0.5)	1 (0.5–1)
Udder T (°C)									
LPS		36.3 (34.7–36.9)	36.6 (34.7–37.4)	36.2 (35–37)	36.9 (36.6–37.2)	36.1 (36–37.5)	36.4 (35.8–37)	36.8 (36–37.3)	36.4 (36–37.2)
LTA		35.1 (34.1–36.6)	34.7 (34–37.4)	35.9 (34.3–37.1)	35.5 (33.8–37.4)	34.4 (33.6–36.8)	35.2 (34.1–37.0)	36.2 (34.3–37.7)	36.9 (33.9–37.6)
Control		33.9 (32.5–35.1)	34 (32.8–35)	34.3 (32.9–35.7)	34.6 (33–35.8)	33.5 (32.9–34.8)	34.4 (33.3–35.5)	33.9 (33.1–35.2)	34.5 (33.5–36)
MNT (N)									
LPS		22.4 (13.4–24.6)	22 (11–24.6)	22.7 (11.8–24.6)	24.6 (22.6–24.6)	18.3 (13.9–23.4)	23.6 (18.2–24.6)	23.9 (19.1–24.6)	21.8 (15.8–24.6)
LTA		24.6 (24.6–24.6)	23.3 (16.6–24.6)	24.6 (19.7–24.6)	24.6 (17.3–24.6)	24.6 (19.4–24.6)	24.6 (21.7–24.6)	23.9 (17.6–24.6)	18.2 (10.7–24.6)
Control		24.6 (23.8–24.6)	24.6 (24.6–24.6)	20.8 (19.2–24.6)	24.6 (22–24.6)	15.5 (11.5–20.6)	22.1 (20.5–23.9)	18.2 (12–23.6)	20.4 (15.1–24)

On the control day, the intramammary challenge, milk and blood sampling were simulated. Medians and interquartile ranges (IQR) at 2, 4, 6 and 8 h after the simulated challenge (control day)/challenge (challenge day) are reported

### Laboratory analysis

**Fig. 4:**
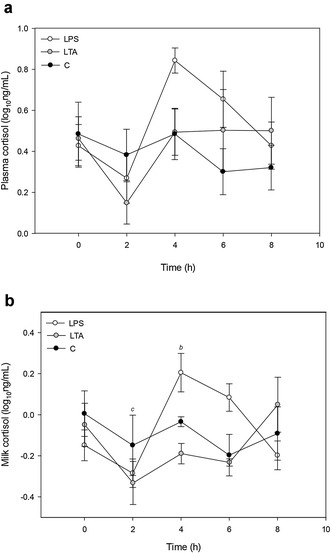
Cortisol concentrations in plasma (**a**) and milk from infused quarters (**b**). Cortisol concentrations in plasma (**a**) and milk from infused quarters (**b**) on the challenge day in the LPS (lipopolysaccharide) (*white circles*), LTA (lipoteichoic acid) (*grey circles*) and control groups (*black circles*). Data are expressed as mean ± SEM. *b* significantly different from t0 (LPS); *c* significantly different from t0 (LTA)

For plasma cortisol, no significant difference among groups was found (Fig. 4a) but a significant time × treatment effect (P = 0.003) was identified for milk cortisol. In the LPS group, 4 h after the challenge, milk cortisol was significantly higher than at t0 (P < 0.001); no quarter effect was detected. In the LTA group, 2 h after the challenge, milk cortisol was significantly lower than at t0 (P < 0.001). No changes over time for milk cortisol were observed in the C group (Fig. 4b).

## Discussion

Aim of this study was to evaluate and compare the effects of an intramammary challenge of LTA or LPS on the degree of pain and discomfort as indicated by physiological and behavioral variables in cows. Our findings indicate that significant alterations of selected outcome variables occurred mostly in LPS treated cows, thus leading to a rejection of the hypothesis that similar degree of pain occurs in both LTA and LPS experimentally induced mastitis.

In the present study, the physiological outcome variables heart rate, respiratory rate and rectal temperature were evaluated. While these parameters have been shown to be good indicators of disease severity in spontaneously occurring mastitis [17, 22], they were not significantly altered by the LPS and LTA treatments, most probably due to the fact that sub-clinical disease levels were targeted.

On the challenge day, all cows spent less time lying than on the control day. Despite the effort to simulate all manipulations foreseen for the challenge day on the control day, the presence of more people and equipment in the barn might have provoked a higher degree of disturbance to the cows, independently from the treatment group. Moreover, as udder and liver biopsies were taken at the end of the control day, it cannot be excluded that residual discomfort in the affected area might have prevented the cows from lying down [23]. For time spent lying contrasting results have been previously reported in cows affected by LPS-induced mastitis: while in some studies a clear reduction of the time spent lying was detected after the intramammary challenge [4, 6], in others no differences were found [24]. This discrepancy might confirm that direct comparison of experimental results from studies evaluating endotoxins-induced mastitis should be performed with caution, as targeted levels of severity, and thus clinical and behavioral consequences, might be different.

Several studies on LPS induced mastitis have reported reduced food intake and reduced time spent ruminating following the intramammary challenge [3, 24]. The peak effect seems to occur 3–9 h after the LPS challenge [24], indicating that the evaluation time of 8 h used in the present study should be adequate to detect challenge-induced alterations if present. While a trend for reduced time spent eating was detected in the LPS group, no differences among groups were found for time spent ruminating. Similar results have been previously reported [7] and, as hypothesized above, they might be linked to the severity degree of the disease. The lack of differences among groups for the postural variable hock-to-hock distance corroborates this hypothesis. Wider hind limb stance is expected to occur in cows affected by mastitis due to udder inflammation: increased hock-to-hock distance was found in mild to moderate clinical mastitis cases [17] in one study while it was unchanged in another [5].

For pain evaluation, a condition-specific multidimensional pain scale was designed. The structure of the scoring system was similar to the one used previously in other species [25] but the included items were meant to be specific for bovine mastitis and whenever possible evidence-based. The first item was a simple numerical rating scale allowing the observer to attribute a general subjective pain score. The second item was a list of possible expected manifestations, the first three being related to facial expression. While facial pain scales have not been validated so far for bovines, it is generally accepted that modifications of facial expression accompanying pain are rather conserved among mammal species [26]. Ears, eyelid and nostrils modifications indicating pain have been described in mice [27], rats [28] and horses [29] and were therefore expected to be potentially modified in cows with experimentally induced mastitis. Restlessness [5], apathy [22] and wide hind limb stance [17] have been previously reported in bovine mastitis. Absence of interactive behavior and response to food were included as general sickness indicators [2], while presence of back arch, reported in bovine affected by lameness [30] and metritis [31], had been observed to occur in clinical mastitis cases and was therefore included in the scoring system. Reactivity to back palpation was expected to potentially occur in painful mastitis as a form of secondary hyperalgesia and muscle hypersensitivity due to postural abnormalities. Additionally, the local items udder edema and reaction to udder palpation were meant to provide disease-specific information as previously reported [22]. Total pain index increased in all treatment groups during the challenge day, with significantly higher values for the LPS group compared to the control group. These findings suggest two considerations. First, a certain treatment-independent day-time effect is detected by the scale, probably representing additional stress provoked by human presence during the challenge day. Second, the total pain index indicates higher degree of pain following the LPS challenge. Similarly, the dynamic-interactive VAS pain scores were mostly affected by the LPS treatment, even if values significantly higher than baseline were found for LTA and control groups at single time points. Visual analogue scales for pain evaluation are daily used in humans [32] and have previously been applied to evaluate pain in ruminants [18, 33]. For veterinary use, dynamic-interactive VAS scales are generally preferred, as the responses to the dynamic interaction with the animal add important information for a correct pain assessment [34]. They are based on subjective assessment of pain following observation and dynamic interaction with the animal. As interobserver variability might be high, it is important to have a single observer throughout the study period when using visual analogue scales. In the present study, total pain index and dynamic-interactive VAS were significantly correlated and led to similar results.

Local signs of disease have been commonly used to evaluate the severity of clinical mastitis [22]. In endotoxins-induced mastitis, rapid influx of neutrophils in the mammary gland occurs [24] accompanied by local signs of inflammation, like edema [7], increased udder temperature [17] and hyperalgesia [35].

In the present study, prevalence of udder edema was higher in the LPS than in the control group. While udder edema has been reported to occur in both experimentally induced and naturally occurring coliform mastitis, it does not appear to be a typical sign in staphylococcal mastitis [36]. Indeed, LTA has been demonstrated to induce a weaker effect on vascular permeability [12] than LPS, possibly corroborating our findings.

Udder temperature of cows in the LPS and LTA groups was higher at some time points after the simulated challenge/challenge than at their first early morning measurement. Since no day effect could be detected, the changes were likely due to treatment-unrelated factors. Udder surface temperature has been shown to vary dependently on circadian oscillations, stage of lactation, environmental temperature and physical activity. In the present study the highest values were measured at 2, 6 and 8 h after the simulated challenge/challenge. Similar findings were previously reported for healthy cows, where a rise in udder temperature was observed between 0900 and 1100 and at late afternoon, with minimal values being recorded between 0400 and 0600 [37].

No differences in udder mechanical nociceptive thresholds were detected in the present study. Contrasting results on nociceptive thresholds have been previously reported for mastitic cows. While nociceptive thresholds to hind limb and udder laser stimulations increased during *E. coli* mastitis indicating hypoalgesia [8], thresholds to thermal hind limb stimulations were lower in cows affected by clinical mastitis compared to healthy controls indicating hyperalgesia [35]. Probably the severity of systemic illness accompanying the inflammation of the mammary gland strongly affects nociceptive thresholds and the reactivity to local stimulation. In presence of severe sickness symptoms like somnolence, lethargy and depression, it is more likely to observe an increase rather a decrease in sensitivity to pain.

While plasma cortisol was not significantly affected by treatment and was within the previously reported range for healthy lactating dairy cows [38], milk cortisol was significantly higher in the LPS group 4 h after the challenge compared to t0. Milk cortisol is considered as a useful indicator of response to acute stressors acting up to 2 h before sampling in lactating dairy cows [39]. As cortisol measured in milk derives from the systemic circulation, higher milk cortisol might reflect a higher permeability of the blood-milk barrier in LPS treated cows. This hypothesis is supported by the finding of higher levels of lactate dehydrogenase in LPS compared to LTA treated cows [14]. In a recent study, significant effects of SCC on milk cortisol were found for SCC above 400 × 10^3^ cells/ml. This finding suggests that hypothalamic–pituitary–adrenal axis activation following an antigenic stimulation can be detected only in case of a severe inflammatory response [40].

The LPS dose of 0.2 µg used in the present study is lower than what generally described in literature [7], while 20 µg LTA has been previously administered to compare LTA and LPS induced immune responses [10]. The highest SCC values, reached approximately 6 h after the challenge, correspond to a subclinical-to-clinical threshold for mastitis. The doses of LPS and LTA were chosen to induce a similar SCC increase as previously reported [41]. Although using other indicators of mastitis severity degree might have led to different results, SCC is a simple quantitative measure used both in clinical and in experimental settings and was considered the most adequate to compare the immune response to this challenge. Interestingly, a high correlation between total pain index and SCC was found, independently from the treatment groups.

Main limitation of the current study is the small size of the treatment groups. As both LPS and LTA experimental models of mastitis are highly standardized and repeatable [41], groups of 6 animals, as determined for the parallel immunological study, were considered to be sufficient for the purpose of this study as well; indeed, statistically significant results could be found even after applying very restrictive Bonferroni corrections. Furthermore, the presence of a small control group in addition to the control day in both LPS and LTA groups, contributed to differentiate between manipulation-induced and treatment-induced changes. Another limitation might be represented by the fact that the cows included were of different breeds and parities. These factors might potentially affect some of the measured outcome variables, like it has been recently described for milk cortisol [40]. Finally, milk and plasma cortisol concentrations, as well as SCC, were not measured on the control day. Therefore, only one pre-challenge baseline value was available for these variables.

## Conclusions

When administered at equipotent doses targeting a standard SCC increase, intramammary LPS seems to be accompanied by a higher degree of pain and discomfort than LTA, as suggested by the modifications of the outcome variables total pain index, dynamic interactive VAS, udder edema and milk cortisol.

## Additional file

**Additional file 1.** Summary of the variables at cow and quarter level used for statistical analysis.
