# Supplementary material for: Experimentally induced subclinical mastitis: are lipopolysaccharide and lipoteichoic acid eliciting similar pain responses?
Source: Acta Vet Scand. 2017 Jun 14;59:40. doi: 10.1186/s13028-017-0306-z (PMC5471899; doi:10.1186/s13028-017-0306-z)
Supplement: Supplementary file 1 — Additional file 1. Summary of the variables at cow and quarter level used for statistical analysis. [file 13028_2017_306_MOESM1_ESM.docx]

**Additional File 1**

Summary of the variables at cow and quarter level used for statistical analysis.

| Level | Variable | Distribution | Correlation structure | Random effect | Number of observations |
| --- | --- | --- | --- | --- | --- |
| Cow-day | Eat time (%, sum) | Normal | cs | - | 30 |
| Cow-day | Rumination time (%, sum) | Normal | vc | - | 30 |
| Cow-day | Lay time  (%, sum) | Normal | vc | - | 30 |
| Cow-hour | Dynamic interactive visual analogue scale | Normal | toep | - | 225 |
| Cow-hour | Total pain index | Negative binomial | - | Cow | 269 |
| Cow-hour | Heart rate | Normal | arma (1,1) | - | 270 |
| Cow-hour | Respiratory rate | Normal | arma (1,1) | - | 270 |
| Cow-hour | Rectal temperature | Normal | arma (1,1) | - | 269 |
| Cow-hour | Intertarsal distance | Normal | arma (1,1) | - | 242 |
| Cow-hour | Plasma cortisol (log_10_) | Normal | vc | - | 75 |
| Quarter-day | Reaction to udder palpation (sum) | Binomial | arma(1,1) | Cow | 60 |
| Quarter-hour | Udder surface temperature | Normal | arma (1,1) | Cow | 475 |
| Quarter-hour | Udder edema | Binomial | arma (1,1) | Cow | 536 |
| Quarter-hour | Nociceptive threshold | Ordinal | - | Quarter + Cow | 202 |
| Quarter-hour | Milk cortisol (log_10_) | Normal | vc | Cow | 127 |
